# *Saposhnikovia divaricata* Inhibits Inflammation, Oxidative Stress, and Ferroptosis to Alleviate DSS-Induced Ulcerative Colitis

**DOI:** 10.3390/antiox15020258

**Published:** 2026-02-18

**Authors:** Lin Liu, Qiting Dou, Xiaoxuan Zhao, Yun Liang, Ziyi Tian, Dantong Su, Lin Zhou, Xuguang Hu

**Affiliations:** School of Chinese Materia Medica, Guangdong Pharmaceutical University, Waihuan East Road, Guangzhou 510006, China; 2112342097@stu.gdpu.edu.cn (L.L.); 2112426046@stu.gdpu.edu.cn (Q.D.); 2112526025@stu.gdpu.edu.cn (X.Z.); 2112348043@stu.gdpu.edu.cn (Y.L.); 2112348088@stu.gdpu.edu.cn (Z.T.); 2112450097@stu.gdpu.edu.cn (D.S.)

**Keywords:** ulcerative colitis, inflammation, oxidative stress, ferroptosis

## Abstract

Ulcerative colitis (UC) is a chronic inflammatory bowel disease that seriously jeopardizes health. *Saposhnikovia divaricata* (FF) has anti-inflammatory and antioxidant pharmacological effects. Numerous studies have demonstrated the efficacy of FF in alleviating UC, but the potential mechanism by which inhibiting ferroptosis alleviates UC remains unclear. This research aims to investigate the ways in which FF regulates inflammation, oxidative stress and ferroptosis to attenuate UC. Firstly, the chemical compounds of FF were identified by UPLC-Q-Orbitrap HRMS. FF significantly reduced levels of nitric oxide (NO), proinflammatory cytokines and reactive oxygen species (ROS) in the lipopolysaccharide (LPS)-induced inflammation in RAW264.7 macrophage cells. We used 3% Dextrose sulfate sodium (DSS) to establish the UC model in C57BL/6 mice. FF (8.4 g/kg) effectively ameliorated the symptoms of weight loss and colon damage, significantly attenuating oxidative stress and modulated the levels of ferroptosis markers in the colon. Moreover, FF can down-regulate the expression of p53 protein and up-regulate the expression of SLC7A11 and GPX4 proteins. The results showed that FF can inhibit inflammation, oxidative stress, and ferroptosis to alleviate DSS-induced UC, by regulating the p53 signaling pathway.

## 1. Introduction

Ulcerative colitis (UC) is a chronic, non-specific inflammatory bowel disease [1]. Its pathogenesis involves a complex interplay of factors [2], including oxidative stress [3], epithelial barrier defects [4], immune cell infiltration [5], and gut microbiome dysbiosis [6]. Clinically, UC is characterized by symptoms such as recurrent diarrhea, abdominal pain, and the frequent presence of blood, mucus, or pus in the stool [7]. The incidence of UC has been rising steadily in recent years [8].

The primary therapeutic goals in UC management are to control inflammation, alleviate symptoms, and improve the patient’s quality of life [9]. Standard pharmacological treatments, including aminosalicylates, glucocorticoids, and immunosuppressants [10], are effective in ameliorating intestinal inflammation. However, these agents are often associated with adverse effects—such as allergic reactions, osteoporosis, and gastrointestinal disturbances [11]—and offer a low rate of lasting cure [12]. Consequently, identifying novel, credible, and effective therapeutic targets remains a critical imperative.

Advances in medical research have deepened the understanding of UC pathogenesis, with recent studies confirming the involvement of ferroptosis in disease progression [13,14,15,16]. Ferroptosis is a novel form of iron-dependent regulated cell death driven by lipid peroxidation [17]. Its biochemical hallmarks include depletion of intracellular glutathione (GSH) [18] and reduced activity of glutathione peroxidase 4 (GPX4), which thereby induce the collection of lipid peroxide [19], and consequent accumulation of lipid peroxides. Furthermore, the accumulation of divalent iron ions (Fe^2+^) catalyzes the Fenton reaction [20], leading to excessive generation of reactive oxygen species (ROS) [21,22]. Similar dysregulation is observed in other oxidative stress markers, such as malondialdehyde (MDA), lipid peroxides (LPO), and superoxide dismutase (SOD).

Oxidative stress and inflammation in UC are intimately linked and mutually reinforcing. Excessive ROS production damages cellular proteins and DNA, triggering the release of pro-inflammatory cytokines such as interleukin-1β (IL-1β), interleukin-6 (IL-6), and tumor necrosis factor-α (TNF-α) [23]. Conversely, the inflammatory response increases oxygen consumption at the site of injury, further promoting ROS accumulation [24]. This interaction creates a self-perpetuating vicious cycle that drives the progression of UC.

Additionally, seminal work by Gu Wei’s team ([25], Nature, 2015) established ferroptosis as a p53-mediated activity in tumor suppression, demonstrating that p53 inhibits cystine uptake by repressing solute carrier family 7 member 11 (SLC7A11) expression. Emerging evidence indicates that p53 also participates in ferroptosis within the context of intestinal diseases [26,27]. SLC7A11 is a critical component of the cystine/glutamate antiporter (system Xc^−^). Its inhibition blocks cystine import, leading to intracellular glutathione (GSH) depletion [28]. This deficiency impairs the activity of glutathione peroxidase 4 (GPX4), thereby compromising cellular antioxidant defenses and rendering cells susceptible to ferroptosis [29].

In recent years, extensive research has revealed that Chinese herbal medicines and their active constituents can modulate ferroptosis and mitigate inflammation through various pathways, offering unique advantages for alleviating UC [30,31,32]. The research by Zhang Jingyan’s team demonstrated that the ameliorative effect of *Scutellaria baicalensis* extracts in UC is closely related to the regulation of lipid peroxidation and GPX4 mediated intestinal epithelial ferroptosis [33]. Li Wenwen’s team found that Lizhong decoction could significantly mitigate DSS-induced UC, and its potential mechanism mainly involved the inhibition of cellular ferroptosis by regulating the Nrf2/SLC7A11/GPX4 pathway to attenuate the oxidative damage [8]. Consequently, investigating TCM-based interventions targeting the ferroptosis process has emerged as a promising and active area of biomedical research.

*Saposhnikovia divaricata* (Fangfeng, FF) is derived from the root of *Saposhnikovia divaricata* (Turcz.) Schischk. Its medicinal application was first documented in Shennong’s Classic of Materia Medica, China’s earliest pharmacological compendium, and it has been widely employed in classical formulations to treat gastrointestinal symptoms in UC patients [34,35]. While several contemporary studies have confirmed the efficacy of FF in treating UC [36,37], its potential mechanism involving the modulation of ferroptosis remains unexplored.

To address this gap, we employed a dextran sulfate sodium (DSS)-induced murine UC model and a lipopolysaccharide (LPS)-induced RAW264.7 macrophage inflammation model to validate the anti-inflammatory and antioxidant effects of FF. Subsequently, we investigated the impact of FF on ferroptosis during UC treatment. Guided by network pharmacology predictions, we conducted experiments to elucidate how FF regulates ferroptosis. Our results demonstrate that FF inhibits ferroptosis via the p53 signaling pathway. By innovatively linking UC pathology with ferroptosis, this study also provides new evidence for the efficacy of FF in UC treatment.

## 2. Materials and Methods

### 2.1. Drugs and Reagents

Lipopolysaccharide (LPS) was purchased from Sigma-Aldrich (St. Louis, MO, USA, Lot #0000223032). Cell culture reagents included DEME high-glucose medium (Gibco, New York, NY, USA, Lot #6125439), fetal bovine serum (FBS) (Pricella, Wuhan, China, Lot #SA240411), and 1% penicillin-streptomycin solution (Solarbio, Beijing, China, Lot #2500040007). Dextran sulfate sodium (DSS) and phosphate-buffered saline (PBS) were obtained from Dalian Meilun Biotechnology Co., Ltd. (Dalian, China, Lot #J0730E and #MA0015, respectively).

The following assay kits were used: nitric oxide (NO) detection kit (Beyotime Biotech Inc., Shanghai, China, Lot #A355250521); Cell Counting Kit-8 (CCK-8) (Dalian Meilun Biotechnology Co., Ltd., Dalian, China, Lot #MA0218); ELISA kits for IL-1β, IL-6, TNF-α, and LPO (Jiangsu Meimian Industrial Co., Ltd., Yancheng, China, Lot #MM-0040M1, #MM-0163M1, #MM-0132M1, #MM-0716M1); and assay kits for Fe^2+^, GSH, SOD, and MDA (Jiangsu Edison Biotechnology Co., Ltd., Nanjing, China, Lot #ADS-W-QT027, #ADS-W-G001, #ADS-W-KY011, #ADS-W-YH002). The fluorescent probes DCFH-DA and DHE were sourced from Beyotime Biotech Inc. (Shanghai, China, Lot #D1206D and #SOO63, respectively).

Primary antibodies against GPX4 (Item #67763-1-Ig) and SLC7A11 (Item #26864-1-AP) were purchased from Proteintech Group Inc. (Chicago, IL, USA). The p53 antibody was obtained from Boster Biological Technology Co., Ltd. (Pleasanton, CA, USA, Item #BM0101). The positive control drug mesalamine (0.25 g/tablet) was supplied by Heilongjiang Tianhong Pharmaceutical Co., Ltd. (Harbin, China, Lot #20231202).

### 2.2. Preparation of Saposhnikovia divaricata (FF) Extract

The crude drug of *Saposhnikovia divaricata* (FF) was purchased from Guangdong Huidakang Pharmaceutical Co., Ltd. (Guangdong, China) and authenticated by Professor Liu Jizhu of the Department of Chinese Medicine Identification at Guangdong Pharmaceutical University. For extract preparation, 30 g of FF crude material were pulverized and subjected to reflux extraction with 300 mL of 70% aqueous ethanol at 40 °C for 4 h. The resulting extract was filtered, and the filtrate was lyophilized to obtain a dry powder, which was stored at −20 °C until use.

### 2.3. Chemical Profiling of FF

For chemical characterization, 1 g of the FF lyophilized powder was ultrasonicated in 20 mL of 80% methanol for 1 h. A 1 mL aliquot of the suspension was then centrifuged at 12,000 rpm and 4 °C for 10 min. Subsequently, 100 µL of the supernatant was diluted with 100 µL of ultrapure water, and the mixture was transferred to an injection vial for UPLC-Q-Orbitrap HRMS analysis.

Chromatographic separation was achieved using an ACQUITY UPLC HSS T3 column (1.7 µm, 2.1 mm × 100 mm) maintained at 40 °C. The mobile phase consisted of water with 0.1% formic acid (phase A) and acetonitrile (phase B), delivered at a flow rate of 0.3 mL/min. The following linear gradient was applied: 0–1 min, 98% A; 1–14 min, 98% to 70% A; 14–25 min, 70% to 0% A; 25–28 min, 0% A; 28–28.1 min, 0% to 98% A; 28.1–29.5 min, 98% A. The injection volume was 6.0 µL.

Mass spectrometric detection was performed using a Q Exactive hybrid quadrupole-Orbitrap mass spectrometer (Thermo Fisher Scientific, Waltham, MA, USA) equipped with a heated electrospray ionization source (HESI-II). The ion source parameters were set as follows: spray voltage, +3.7 kV (positive) and −3.5 kV (negative); capillary temperature, 320 °C; sheath gas pressure, 30 psi; auxiliary gas pressure, 10 psi; and auxiliary gas heater temperature, 300 °C.

### 2.4. Cells Culture

RAW264.7 murine macrophages were obtained from iCell Bioscience Inc. (Shanghai, China; Item No. iCell-m047). The cells were maintained in DMEM high-glucose medium, supplemented with 10% fetal bovine serum and 1% penicillin-streptomycin, at 37 °C in a humidified atmosphere containing 5% CO_2_.

### 2.5. Viability Assay

Stock solutions of LPS (1 µg/mL) and FF (50, 100, and 200 µg/mL) were prepared in PBS and sterilized by filtration through a 0.22 µm membrane (PES Membrane Filter, Millipore, St. Louis, MO, USA). Cells were seeded in a 96-well plate and allowed to adhere overnight. The medium was then replaced with fresh medium containing the indicated concentrations of FF and LPS (1 µg/mL). Following a 24 h incubation, the medium was removed, and 100 µL of 10% CCK-8 solution (in serum-free medium) was added to each well. The plate was incubated in the dark for 2 h, after which the absorbance at 450 nm was measured using a microplate reader.

### 2.6. Detecting the Levels of Nitric Oxide (NO) and Inflammation Cytokine

Cells were seeded into a 6-well plate and cultured overnight. Prior to LPS stimulation, cells were pretreated with FF for 1 h. Subsequently, cells were exposed to LPS (1 µg/mL) for 24 h. Following incubation, the culture supernatant was collected. Levels of nitric oxide (NO) were quantified using the Griess reagent method, while concentrations of proinflammatory cytokines (IL-1β, IL-6, TNF-α) were measured using commercial ELISA kits according to the manufacturers’ instructions.

### 2.7. Intracellular ROS Assay

Cells were cultured and treated as described in Section 2.6. Following treatment, cells were washed twice with PBS and incubated with the fluorescent probe DCFH-DA (10 µM) in serum-free medium at 37 °C in the dark for 20 min, according to the manufacturer’s protocol. After incubation, cells were washed twice with PBS to remove excess probe. Intracellular reactive oxygen species (ROS) levels were determined by measuring fluorescence intensity with a microplate reader at an excitation wavelength of 485 nm and an emission wavelength of 535 nm.

### 2.8. Animal Experiments

Male C57BL/6 mice (six weeks old, 18–22 g) were sourced from Zhiyuan Biotechnology Co., Ltd. (Guangdong, China) and maintained under specific pathogen-free (SPF) conditions in the Animal Center of Guangdong Pharmaceutical University. All animal procedures were approved by the Animal Ethics Committee of Guangdong Pharmaceutical University (License No.: gdpulacspf2022596) and conducted in accordance with the National Institutes of Health Guide for the Care and Use of Laboratory Animals.

The starting dose of FF (2.1 g/kg) was derived from the human equivalent dose per the Chinese Pharmacopoeia, with conversion based on FDA guidelines. A safety assessment was conducted using ten graded doses (1.1, 2.1, 3.2, 4.2, 5.3, 6.3, 7.4, 8.4, 9.5, and 10.5 g/kg) according to the method of Li Yue et al. [38]. The maximum tolerated dose was identified as 8.4 g/kg. Based on this finding, the low, medium, and high experimental doses were set at 2.1, 4.2, and 8.4 g/kg, respectively.

Using a randomized block design, mice were divided into six groups (*n* = 10 per group): control (CON), DSS model (DSS), low-dose FF (DSS + 2.1 g/kg FF, FF-L), medium-dose FF (DSS + 4.2 g/kg FF, FF-M), high-dose FF (DSS + 8.4 g/kg FF, FF-H), and positive control mesalamine (DSS + 0.62 g/kg mesalamine, ME).

All mice received a 7-day pretreatment period prior to DSS induction. Treatment groups were administered the corresponding drug doses daily via oral gavage, while CON and DSS groups received an equal volume of 0.9% saline. Starting on day 8, all groups except CON received 3% DSS dissolved in drinking water for 7 days to induce colitis. During this period, drug or vehicle administration continued as described. On day 15, mice were euthanized under anesthesia. Blood samples were collected for serum isolation, and tissue specimens (spleen, kidney, liver, thymus, and colon) were harvested for subsequent analysis.

### 2.9. Assessment of Normal Situation

Following the daily recording of food intake, body weight, and fecal characteristics (consistency and occult blood), the Disease Activity Index (DAI) was calculated according to the established scoring criteria (Table 1) adapted from Xie, Q et al. [2]. The DAI score represents the sum of three component subscores.

### 2.10. Assessment of Colon Injury

Following measurement, colons were longitudinally incised, gently flushed with cold PBS, and macroscopically assessed. The Colon Macroscopic Damage Index (CMDI) was scored according to the established criteria (Table 2) adapted from Xie, Q et al. [2].

### 2.11. Hematoxylin–Eosin (HE) Staining

A 1 cm segment of the distal colon was fixed in 4% paraformaldehyde for 24 h. Tissues were then dehydrated through a graded ethanol series, embedded in paraffin, and sectioned at a thickness of 5 µm. Sections were stained with hematoxylin and eosin (H&E) and imaged under a light microscope for histological analysis.

### 2.12. Assessment of Organ Index

The organ index (organ weight relative to body weight) is a recognized indicator of systemic inflammation [39]. To assess inflammatory status, the spleen, kidneys, liver, and thymus were harvested and weighed. Their respective organ indexes were calculated using the formula: Organ Index (mg/g) = [Organ Weight (mg) / Body Weight (g)].

### 2.13. Detecting the Levels of Cytokines in the Serum

Blood samples were centrifuged at 1165× *g* for 10 min at 4 °C to obtain serum. The concentrations of IL-6, IL-1β, and TNF-α in the serum were quantified using commercial ELISA kits according to the manufacturers’ instructions.

### 2.14. Detecting the Levels of Oxidative Stress and Ferroptosis Markers in the Colon

Colon tissue homogenates were prepared in cold PBS and centrifuged at 855× *g* for 20 min at 4 °C. The resulting supernatant was collected, and the levels of glutathione (GSH), malondialdehyde (MDA), superoxide dismutase (SOD), lipid peroxides (LPO), and ferrous iron ions (Fe^2+^) were quantified using their respective commercial assay kits according to the manufacturers’ protocols.

### 2.15. Immunofluorescence (IF)

Fresh colon tissues were embedded in optimal cutting temperature (OCT) compound and immediately sectioned at a thickness of 10 μm using a cryostat. Sections were washed with PBS and subsequently incubated with the fluorescent probe dihydroethidium (DHE) for 1 h at 37 °C in the dark. Images were then acquired using a fluorescence microscope.

### 2.16. Network Pharmacology Analysis

The active ingredients of FF were collected from the TCMSP database (https://tcmsp-e.com/), applying screening for Oral bioavailability (OB) ≥ 30% and Drug-likeness (DL) ≥ 0.18 values. Potential targets of these compounds were predicted using the Swiss Target Prediction database (https://swisstargetprediction.ch/) and Batman-TCM database (https://ngdc.cncb.ac.cn/) based on Probability > 0.

Disease-related targets for ulcerative colitis (UC) and ferroptosis were collected from the TTD database (https://ttd.idrblab.cn/), OMIM database (https://www.omim.org/) and Gene Cards database (https://www.genecards.org/) using “ulcerative colitis” and “ferroptosis” as keyword. An intersection analysis of the drug and disease targets was performed using Venny2.1.0 (https://bioinfogp.cnb.csic.es/tools/venny/index.html).

The common targets were imported into the STRING database (https://cn.string-db.org/) to construct a protein–protein interaction (PPI) network, with the organism limited to Homo sapiens. The network was analyzed and visualized using Cytoscape software (version 3.10.0). Core targets within the network were identified based on their degree centrality, with node size and color depth representing the degree value.

Functional enrichment analysis of the common targets was performed using the DAVID bioinformatics platform (https://davidbioinformatics.nih.gov/). The top 10 enriched Gene Ontology (GO) terms and the top 20 Kyoto Encyclopedia of Genes and Genomes (KEGG) pathways were selected based on *p*-value. The results were visualized as a bar chart (GO) and a Sankey bubble diagram (KEGG), respectively.

### 2.17. Molecular Docking

The three-dimensional structures of the target proteins were obtained from the RCSB Protein Data Bank (https://www.rcsb.org/). The chemical structures of the top five active compounds, ranked by degree value in the network analysis, were retrieved from the PubChem database (https://pubchem.ncbi.nlm.nih.gov/). Molecular docking simulations were performed using AutoDock Vina (version 1.2.5) to calculate the minimum binding free energy (ΔG, kcal/mol) between each compound and its respective target protein. The docking results were visualized using PyMOL software Version 3.1 to analyze the binding conformations and interactions.

### 2.18. Immunohistochemistry (IHC)

Paraffin-embedded colon sections were prepared as described previously. For immunohistochemical analysis, sections were incubated overnight at 4 °C with the following primary antibodies diluted in blocking buffer: anti-p53 (1:200), anti-SLC7A11 (1:200), and anti-GPX4 (1:200). After washing with PBS, sections were incubated with appropriate horseradish peroxidase (HRP)-conjugated secondary antibodies at 37 °C for 1 h. Antigen visualization was performed using 3,3′-diaminobenzidine (DAB) as a chromogen, and images were captured under a light microscope.

### 2.19. Statistical Analysis

All statistical analyses were performed using GraphPad Prism software (version 10). Data normality and homogeneity of variance were assessed using the Shapiro–Wilk test and Levene’s test, respectively. Body weight change, food intake, water intake, and Disease Activity Index (DAI) scores were analyzed by two-way analysis of variance (ANOVA). All other data were analyzed by one-way ANOVA. For all analyses, a *p*-value of less than 0.05 was considered statistically significant.

## 3. Results

### 3.1. Chemical Characterization of Saposhnikovia divaricata (FF)

**Figure 1:**
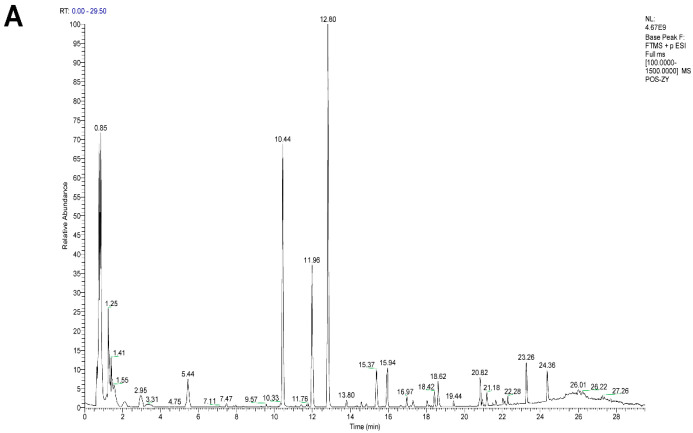

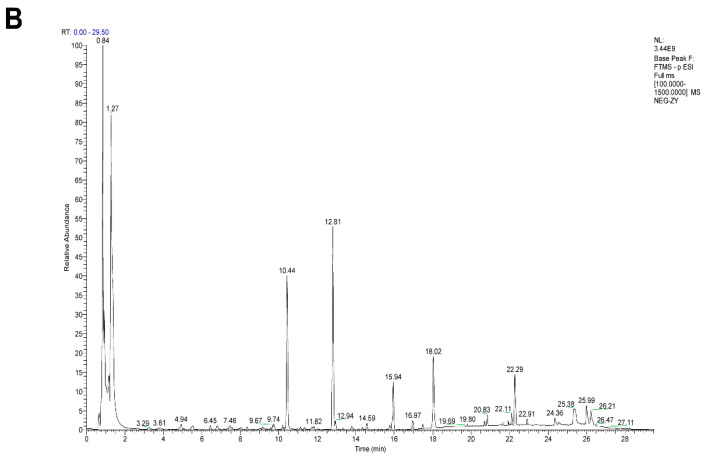
UPLC-Q-Orbitrap HRMS analysis of the extract of FF (**A**) positive ion modes. (**B**) negative ion modes.

UPLC-Q-Orbitrap HRMS analysis identified 23 chemical components in the *Saposhnikovia divaricata* (FF) extract based on retention time and mass spectrometric data (representative chromatogram shown in Figure 1). These included compounds such as 5-O-Methylvisammioside, Prim-O-glucosylcimifugin, Sec-O-glucosylhamaudol, Decursin, 4-O-Feruloylquinic acid, Isoimperatorin and Imperatorin (Table 3). The identified chemical profile is consistent with the quality standards outlined in the Chinese Pharmacopoeia.

### 3.2. FF Reduced the Levels of NO, Inflammatory Cytokines and ROS in RAW264.7 Cells

**Figure 2:**
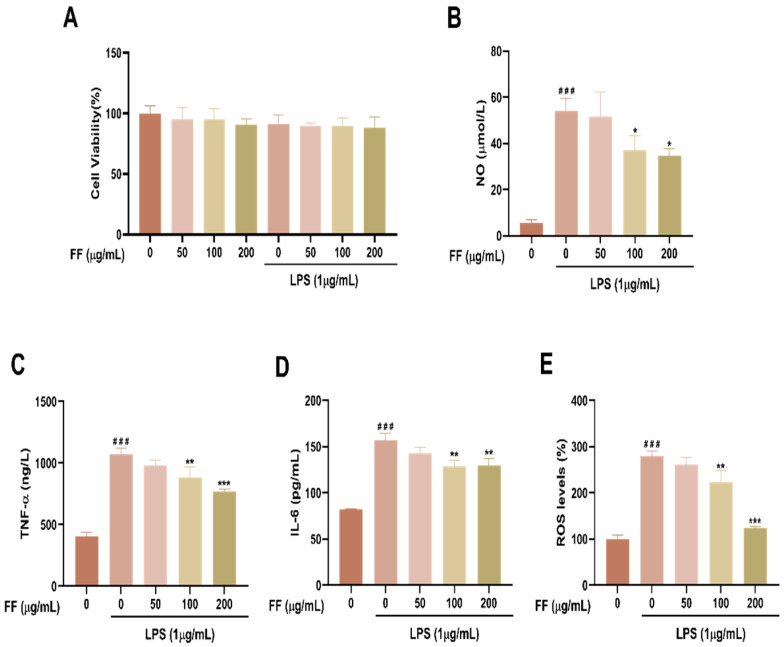
Effect of FF on inflammation and oxidative stress in LPS-stimulated RAW264.7 cells. (**A**) Cell viability following treatment with FF alone or in combination with LPS. (**B**) Nitric oxide (NO) levels. (**C**) Tumor necrosis factor-alpha (TNF-α) levels. (**D**) Interleukin-6 (IL-6) levels. (**E**) Intracellular reactive oxygen species (ROS) levels. Data are presented as the mean ± standard deviation (SD) (*n* = 3 independent experiments, each performed in triplicate). ^###^ *p* < 0.001 versus the control group; * *p* < 0.05, ** *p* < 0.01, *** *p* < 0.001 versus the LPS-treated group.

The CCK-8 assay indicated that neither LPS nor any of the three tested concentrations of FF significantly affected RAW264.7 cell viability, confirming the absence of cytotoxicity within this concentration range (Figure 2A). Nitric oxide (NO) is a well-established marker of cellular inflammation [40]. In LPS-stimulated cells, levels of NO, TNF-α, and IL-6 were significantly elevated compared to the control group (*p* < 0.001; Figure 2B–D), confirming the successful establishment of an inflammatory model. Treatment with FF dose-dependently inhibited the release of these inflammatory mediators. Specifically, the high concentration of FF (200 µg/mL) exerted a stronger suppressive effect on NO, TNF-α, and IL-6 levels than the lower concentrations (*p* < 0.05, *p* < 0.001, *p* < 0.01; Figure 2B–D), demonstrating the anti-inflammatory activity of FF in LPS-induced RAW264.7 macrophages.

Inflammation is closely associated with oxidative stress. LPS stimulation significantly increased intracellular ROS levels, which were more than double those in the control cells (*p* < 0.001; Figure 2E). This oxidative burst was markedly attenuated by treatment with FF at 100 µg/mL and 200 µg/mL (*p* < 0.01 and *p* < 0.001, respectively; Figure 2E). Collectively, these results demonstrate that FF effectively alleviates both inflammation and oxidative stress in LPS-stimulated RAW264.7 macrophages.

### 3.3. FF Improved Symptoms of UC

**Figure 3:**
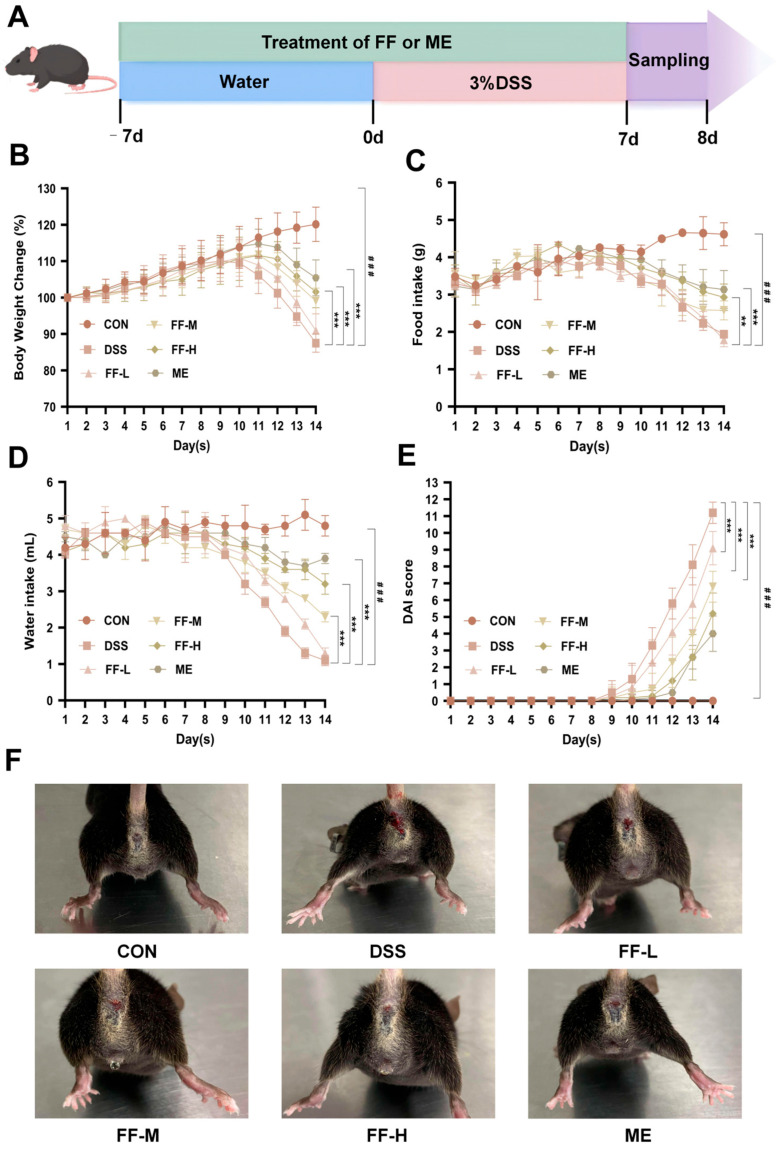
FF treatment alleviates DSS-induced colitis. (**A**) Experimental flow chart. (**B**) Body weight changes. (**C**) Food intake. (**D**) Water intake. (**E**) DAI score. (**F**) Bloody stools. Data are presented as mean ± SD (*n* = 10). ^###^ *p* < 0.001 (vs. the CON group); ** *p* < 0.01, *** *p* < 0.001 (vs. the DSS group).

To evaluate the therapeutic effect of FF on ulcerative colitis (UC), a 14-day drug administration protocol was implemented in a dextran sulfate sodium (DSS)-induced murine model, with sample collection on day 15 (Figure 3A).

Mice in the DSS group exhibited significant body weight loss compared to the control (CON) group (*p* < 0.001, Figure 3B). They also showed markedly reduced food and water intake during the disease induction phase (*p* < 0.001, Figure 3C, D). Concurrently, these mice developed characteristic symptoms of colitis, including diarrhea and hematochezia, leading to a progressive increase in the disease activity index (DAI) score (*p* < 0.001; Figure 3E,F).

Treatment with FF significantly attenuated these clinical manifestations. Compared to the DSS group, FF-administered mice showed mitigated body weight loss, improved food and water consumption, and a reduction in bloody stools, collectively resulting in a lower DAI score (Figure 3B–F).

### 3.4. FF Reduced Colon Injury

**Figure 4:**
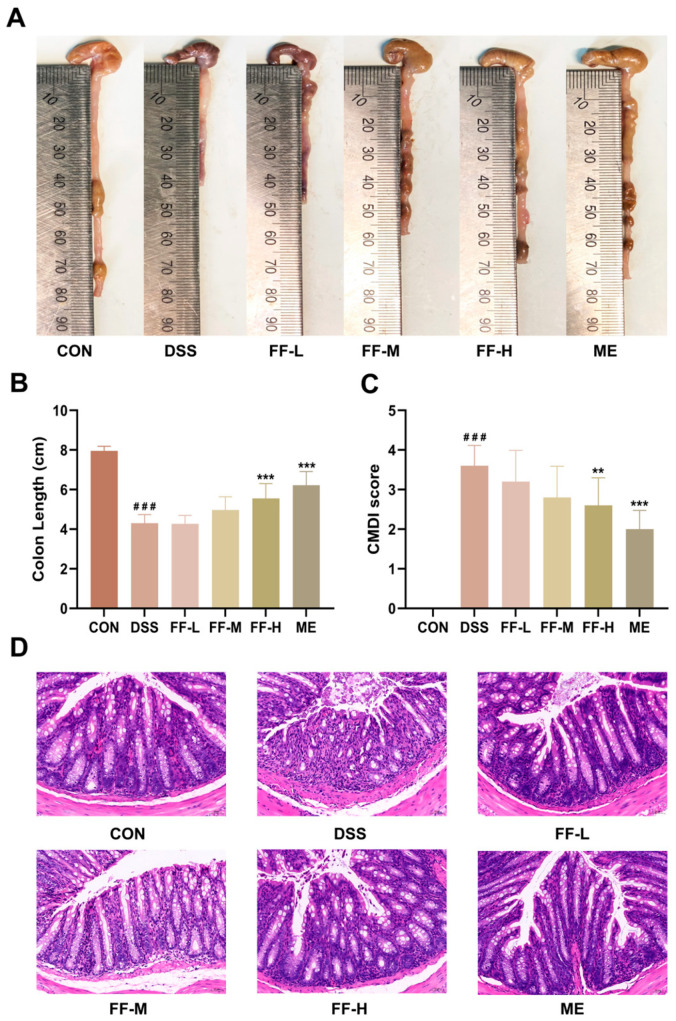
FF ameliorates colon damage in DSS-induced colitis. (**A**) Representative macroscopic images of colons from each experimental group. (**B**) Quantitative analysis of colon length. (**C**) Colon Macroscopic Damage Index (CMDI) scores. (**D**) Representative hematoxylin and eosin (H&E)-stained colon sections (scale bar = 50 μm). Data are presented as the mean ± SD (*n* = 10 mice per group). ^###^ *p* < 0.001 versus the control (CON) group; ** *p* < 0.01, *** *p* < 0.001 versus the DSS model group.

Mice in the DSS group exhibited markedly shortened colons with severe macroscopic damage, including hematoma, edema, mucosal necrosis, and ulceration, compared to the control (CON) group. This was reflected in a significantly higher Colon Macroscopic Damage Index (CMDI) score (*p* < 0.001; Figure 4C). Histological assessment by hematoxylin and eosin (H&E) staining confirmed extensive injury, characterized by a reduced number of intestinal glands, significant inflammatory cell infiltration (predominantly lymphocytes), and severe submucosal edema.

Treatment with FF, particularly at the high dose (FF-H), effectively ameliorated these pathological changes. The FF-H group showed significantly greater colon length (*p* < 0.001; Figure 4A,B) and a lower CMDI score (*p* < 0.01; Figure 4C) compared to the DSS model group. Histological analysis revealed a dose-dependent improvement in tissue architecture, with reduced edema, diminished inflammatory infiltration, and partial restoration of glandular structure (Figure 4D). These results demonstrate that FF alleviates colon injury in DSS-induced colitis, with the most pronounced therapeutic effect observed at the highest dose (FF-H).

### 3.5. FF Reduces the Organ Indexes of the Liver, Kidneys, and Spleen, and Increases the Thymus Index

**Figure 5:**
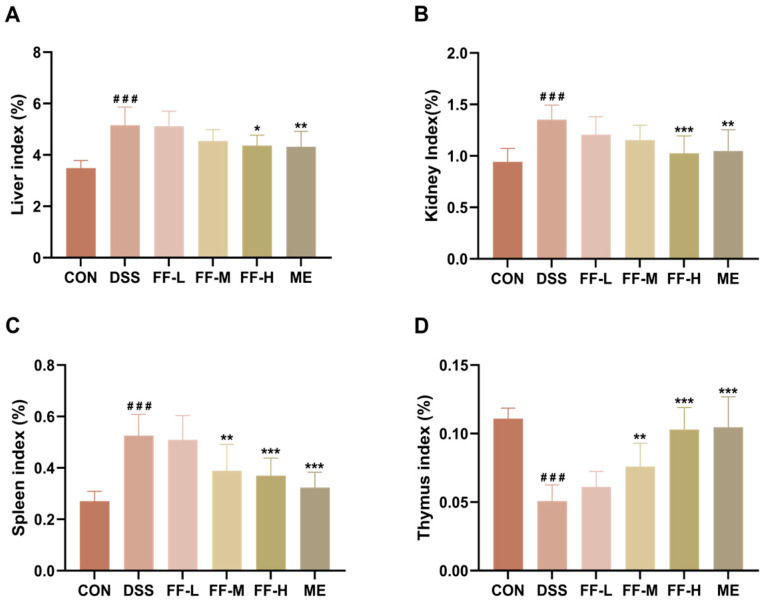
Organ indexes. (**A**) Liver index. (**B**) Kidney index. (**C**) Spleen index. (**D**) Thymus index. Data are presented as mean ± SD (*n* = 10). ^###^ *p* < 0.001 (vs. the CON group); * *p* < 0.05, ** *p* < 0.01, *** *p* < 0.001 (vs. the DSS group).

Compared to the control (CON) group, mice in the DSS model group exhibited significantly elevated organ indices for the liver, kidney, and spleen, along with a decreased thymus index (*p* < 0.001 for all; Figure 5A–D). These alterations are indicative of systemic inflammation and associated organ involvement in colitis.

Treatment with FF effectively reversed these changes. Administration of FF, particularly at the high dose (FF-H), significantly ameliorated the DSS-induced alterations in all four organ indices (Figure 5A–D). Notably, the effect of FF-H on the liver index was comparable to that of the positive control mesalamine (ME) group (*p* < 0.05). Furthermore, the therapeutic efficacy of the FF-H group was significantly superior to that of the low- (FF-L) and medium-dose (FF-M) groups for the kidney, spleen, and thymus indices (*p* < 0.001), demonstrating a clear dose-dependent response.

### 3.6. FF Reduced the Levels of Inflammatory Cytokines in Serum

**Figure 6:**
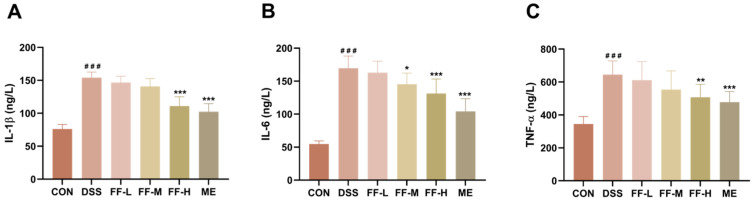
Inflammatory Cytokines in Serum. (**A**) The level of IL-1β. (**B**) The level of IL-6. (**C**) The level of TNF-α. Datas are presented as mean ± SD (*n* = 10). ^###^ *p* < 0.001 (vs. the CON group); * *p* < 0.05, ** *p* < 0.01, *** *p* < 0.001 (vs. the DSS group).

Induction of colitis with DSS resulted in a significant increase in serum levels of the pro-inflammatory cytokines IL-1β, IL-6, and TNF-α compared to the control group (*p* < 0.001; Figure 6A–C). Treatment with FF significantly attenuated this cytokine surge in a dose-dependent manner. Administration of FF at all tested concentrations led to a marked reduction in the levels of IL-1β, IL-6, and TNF-α, with the high-dose FF group (FF-H) demonstrating the most potent anti-inflammatory effect (Figure 6A–C). These results confirm that FF alleviates systemic inflammation in DSS-induced colitis.

### 3.7. FF Inhibited Oxidative Stress and Ferroptosis

**Figure 7:**
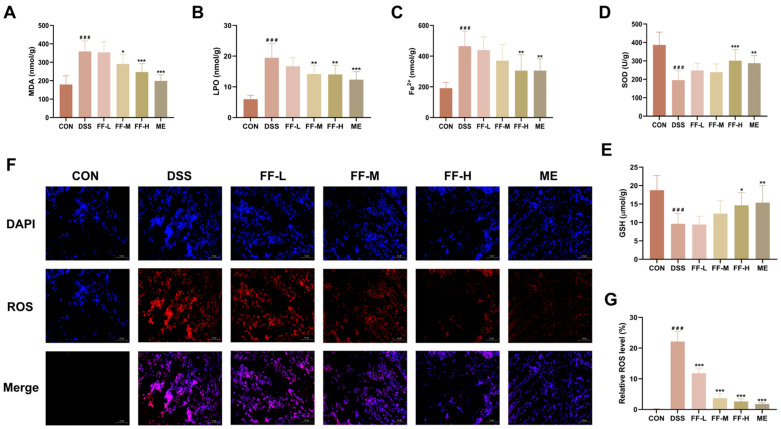
FF alleviates oxidative stress and suppresses ferroptosis in DSS-induced colitis. (**A**) Malondialdehyde (MDA) level. (**B**) Lipid peroxide (LPO) level. (**C**) Ferrous iron (Fe^2+^) level. (**D**) Superoxide dismutase (SOD) activity. (**E**) Glutathione (GSH) level. (**F**) Representative immunofluorescence (IF) images showing reactive oxygen species (ROS) in colon tissues (scale bar = 50 μm). (**G**) Quantitative analysis of relative ROS fluorescence intensity. Data are presented as the mean ± SD (*n* = 10 mice per group for A-E; *n* = 3 independent experiments performed in triplicate for G). ^###^ *p* < 0.001 versus the control (CON) group; * *p* < 0.05, ** *p* < 0.01, *** *p* < 0.001 versus the DSS model group.

Biochemical analysis of colon tissue revealed that DSS induction significantly elevated the levels of oxidative stress and ferroptosis markers, including malondialdehyde (MDA), lipid peroxides (LPO), and ferrous iron (Fe^2+^), while markedly depleting the antioxidants superoxide dismutase (SOD) and glutathione (GSH) (*p* < 0.001 for all; Figure 7A–E).

Treatment with FF dose-dependently reversed these pathological alterations. All FF-treated groups showed significant amelioration of the DSS-induced changes, with the high-dose group (FF-H) exhibiting the most pronounced effect, significantly outperforming the low- (FF-L) and medium-dose (FF-M) groups (Figure 7A–E).

This suppression of oxidative stress was further corroborated by immunofluorescence (IF) staining, which demonstrated a significant increase in colonic reactive oxygen species (ROS) levels in the DSS group (*p* < 0.001). FF treatment, particularly at the high dose, effectively reduced this ROS accumulation (*p* < 0.001; Figure 7F,G).

Collectively, these findings demonstrate that FF effectively inhibits oxidative stress and attenuates ferroptosis in the colon of mice with DSS-induced colitis.

### 3.8. P53 Is a Key Target Through Which FF Alleviates UC by Regulating Ferroptosis

**Figure 8:**
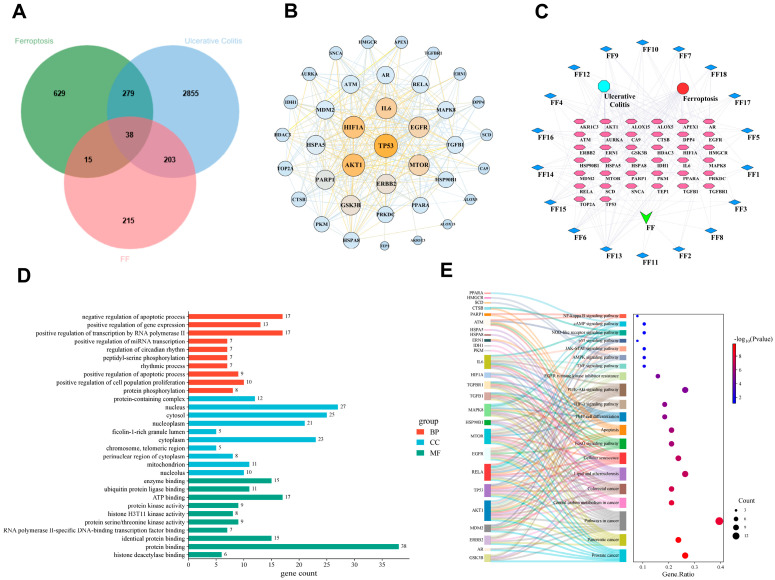
Network pharmacology analysis identifies p53 as a core ferroptosis-related target of FF in UC. (**A**) Venn diagram illustrating the intersection of FF compound targets with ferroptosis- and UC-related targets. (**B**) Protein–protein interaction (PPI) network of the common targets. Node size and color intensity represent the degree of connectivity, with TP53 (p53) identified as the core target. (**C**) Compound-target-pathway network diagram. The inner pink nodes represent the 38 intersecting targets linking FF, UC, and ferroptosis. (**D**) Gene Ontology (GO) enrichment analysis of the common targets, categorized into Biological Process (BP, red), Cellular Component (CC, green), and Molecular Function (MF, blue). (**E**) Kyoto Encyclopedia of Genes and Genomes (KEGG) pathway enrichment analysis.

The animal experiments demonstrated that FF alleviates UC, potentially through the inhibition of ferroptosis. To further elucidate the underlying mechanism, a network pharmacology approach was employed. Database screening identified 18 major active compounds in FF. Intersection analysis of the predicted targets for these compounds and known UC- and ferroptosis-related disease targets yielded 38 common targets, visualized in a Venny diagram (Figure 8A). These common targets were used to construct a protein–protein interaction (PPI) network via the STRING database. Analysis of network centrality identified p53 as the target with the highest degree value, indicated by its prominent node size and color in the PPI visualization (Figure 8B). The compound-target network was further analyzed and visualized using Cytoscape (Figure 8C), and the top 10 key compounds were ranked based on degree centrality (Table 4). Functional enrichment analysis was performed using Gene Ontology (GO) and the Kyoto Encyclopedia of Genes and Genomes (KEGG). The top 10 enriched terms for biological processes (BP), cellular components (CC), and molecular functions (MF) are presented in Figure 8D. KEGG pathway analysis identified the top 20 enriched pathways (Figure 8E). Enrichment in pathways related to HIF-1, PI3K-Akt, TNF, p53, and NF-κB signaling suggests that FF alleviates UC through multi-target regulation of inflammation, immune response, ferroptosis, and metabolic processes.

### 3.9. Molecular Docking

**Figure 9:**
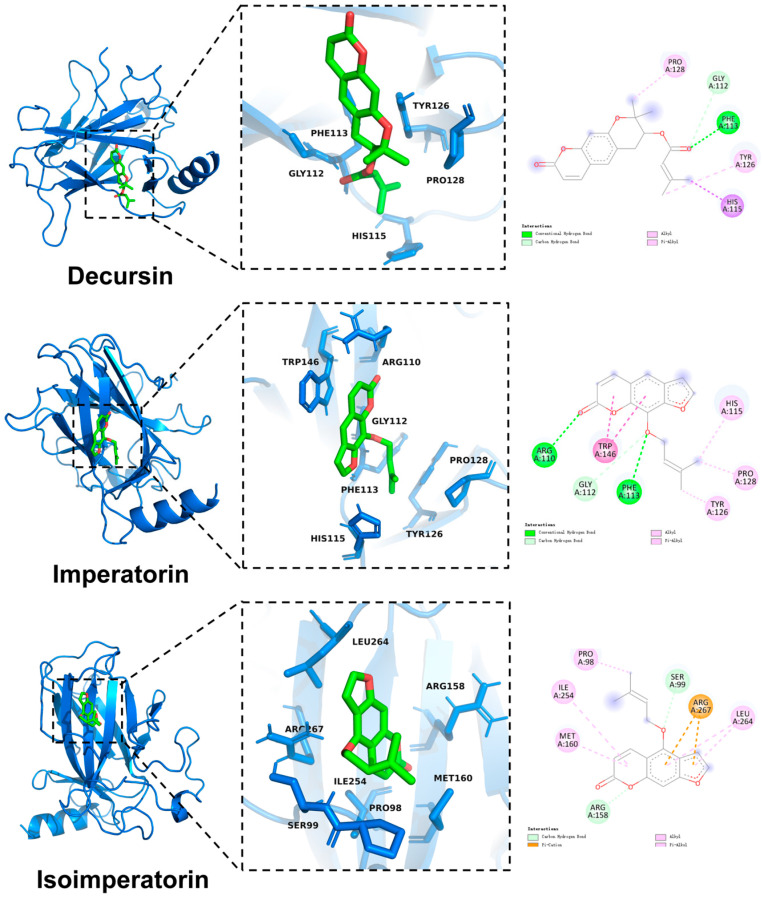
The magnitude of the binding free energy for molecular docking.

To validate the potential interaction between the active ingredients and the predicted core target p53, molecular docking simulations were performed. The top-ranked compounds from the network pharmacology analysis were docked against the p53 protein structure. The minimum binding free energy (ΔG) for each complex was calculated using AutoDock Vina (Figure 9). A binding free energy lower than −5.0 kcal/mol is generally considered indicative of good binding affinity. The results predicted that Decursin, Imperatorin, and Isoimperatorin possess the strongest binding affinities for p53, as shown by their favorable ΔG values (Table 5). Moreover, these three components were effectively detected in our mass spectrometry report.

### 3.10. FF Alleviate UC by Regulating the p53 Signaling Pathway

**Figure 10:**
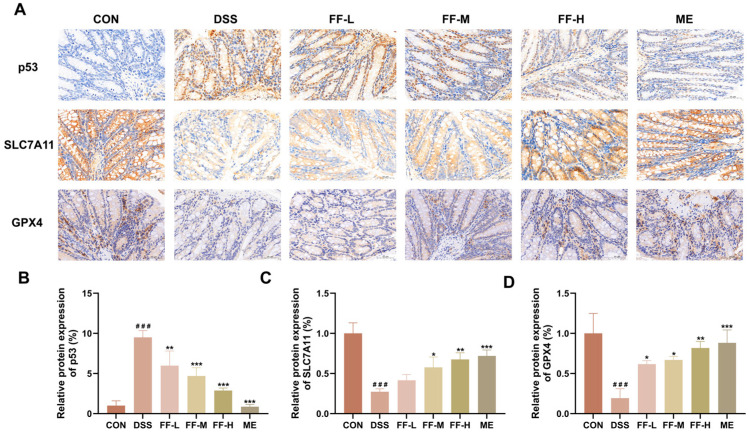
FF modulates the expression of ferroptosis-related proteins in colon tissue via the p53 pathway. (**A**) Representative immunohistochemical (IHC) images of p53, SLC7A11, and GPX4 expression in colon sections (scale bar = 50 μm). (**B**–**D**) Quantitative analysis of the relative protein expression levels of p53 (B), SLC7A11 (C), and GPX4 (D). Data are presented as the mean ± SD (*n* = 3 independent experiments). ^###^ *p* < 0.001 versus the control (CON) group; * *p* < 0.05, ** *p* < 0.01, *** *p* < 0.001 versus the DSS model group.

Immunohistochemical (IHC) analysis confirmed the upregulation of key ferroptosis-related proteins in colon tissue. Mice in the DSS model group exhibited a significant increase in p53 protein expression alongside marked decreases in SLC7A11 and GPX4 protein levels compared to the control group (*p* < 0.001 for all; Figure 10A–D).

Treatment with the high dose of FF (FF-H) effectively reversed these alterations. The FF-H group showed significantly reduced p53 expression and restored levels of SLC7A11 and GPX4 proteins (*p* < 0.001, *p* < 0.01, *p* < 0.01, respectively; Figure 10A–D). These findings suggest that the protective effect of FF against ferroptosis in colitis involves modulation of the p53/SLC7A11/GPX4 signaling axis.

## 4. Discussion

Ulcerative colitis (UC) is characterized by chronic, relapsing inflammation of the colonic mucosa [41]. Its primary clinical manifestations include abdominal pain, diarrhea, and, in many cases, hematochezia [42]. Without effective long-term management, UC is associated with an increased risk of colorectal cancer. Current first-line pharmacological therapies, while effective for inducing and maintaining remission, are often limited by significant adverse effects [43,44,45]. Aminosalicylates commonly induce gastrointestinal disturbances and neurological reactions [46]. Glucocorticoid use is associated with adverse metabolic effects such as centripetal obesity, diabetes mellitus, and osteoporosis, as well as complications like hypertension and peptic ulcers [47,48]. Immunosuppressants carry risks of bone marrow suppression, leading to immunodeficiency and heightened susceptibility to infections.

Traditional Chinese Medicine (TCM) is a comprehensive medical system with a long historical and theoretical foundation. Chinese herbal medicines are often associated with proven therapeutic efficacy and favorable safety profiles, leading to good patient compliance. The major bioactive components of *Saposhnikovia divaricata* (Fangfeng, FF) are coumarins and chromones [49]. Specific coumarins such as decursin, imperatorin, and isoimperatorin can modulate the p53/SLC7A11/GPX4 pathway to exert anti-ferroptosis effects [50,51,52]. These findings are consistent with the established anti-inflammatory and antioxidant pharmacology of FF, which may synergistically contribute to its therapeutic potential [53,54]. *Saposhnikovia divaricata* (FF) is a key component in numerous classical formulas renowned for treating dysentery and diarrhea [55]. Modern research has confirmed that FF alleviates colonic mucosal inflammation, promotes ulcer healing, and mitigates UC symptoms [36]. However, prior investigations have primarily focused on its anti-inflammatory and intestinal barrier-protective properties, leaving its potential role in modulating ferroptosis unexplored.

In the present study, we implemented a 7-day preventive treatment protocol to mirror clinical strategies for managing disease flares. Our results confirm that FF effectively ameliorates hallmark symptoms of DSS-induced colitis, including body weight loss, hematochezia, colon shortening, and histological damage. Extending beyond these phenotypic observations, we further investigated the underlying mechanisms and demonstrated that FF exerts potent antioxidant and anti-ferroptosis effects in this model.

Oxidative stress is a pivotal driver of inflammation in inflammatory bowel disease (IBD) [56]. It disrupts cellular redox balance, exacerbating intestinal damage and perpetuating the inflammatory response [57]. In the inflamed IBD mucosa, infiltrating immune cells release abundant pro-inflammatory cytokines [47,58], while epithelial and immune cells accumulate excessive reactive oxygen species (ROS) [59,60]. This ROS overload inflicts damage on cellular proteins, lipids, and DNA [61]. Concurrently, elevated intracellular ferrous iron (Fe^2+^) catalyzes lipid peroxidation, activating the ferroptosis cascade [62]. These processes create a vicious cycle of escalating cellular injury and disrupted intestinal homeostasis, which aggravates IBD symptoms [63]. Our in vitro experiments demonstrated that FF significantly reduced levels of nitric oxide (NO), pro-inflammatory cytokines (TNF-α, IL-6), and ROS in LPS-stimulated RAW264.7 macrophages, confirming its anti-inflammatory and antioxidant properties at the cellular level. Consistently, in the DSS-induced colitis model, FF treatment lowered serum concentrations of inflammatory cytokines and reduced colonic ROS accumulation, validating these effects in vivo.

Further analysis revealed that FF administration also significantly inhibited the accumulation of Fe^2+^ in colon tissue. This finding, alongside the reduction in oxidative stress markers, provides preliminary evidence that the therapeutic mechanism of FF involves the suppression of ferroptosis.

Ferroptosis is a form of regulated cell death defined by iron-dependent lipid peroxidation [64]. Glutathione peroxidase 4 (GPX4) serves as a crucial cellular antioxidant enzyme, utilizing reduced glutathione (GSH) as a cofactor to reduce lipid hydroperoxides, thereby protecting cell membranes from oxidative damage [65]. During ferroptosis, GSH depletion leads to the functional inactivation of GPX4 [66,67]. The resultant loss of antioxidant defense renders polyunsaturated fatty acids within phospholipid membranes vulnerable to oxidation, leading to the massive generation of lipid peroxides (LPO) [68,69]. These primary peroxidation products decompose into secondary reactive aldehydes, including malondialdehyde (MDA) [70,71]. Superoxide dismutase (SOD), another key antioxidant enzyme, mitigates oxidative stress by catalyzing the dismutation of superoxide anions [72,73]. A decline in SOD activity impairs the clearance of reactive oxygen species (ROS), disrupting cellular redox homeostasis and promoting the ferroptosis cascade. Consequently, MDA, SOD, LPO, ROS, and GSH are established biomarkers for assessing lipid peroxidation and ferroptosis.

In the present study, we observed that FF treatment significantly modulated these classic ferroptosis markers. Specifically, FF reduced the levels of MDA, LPO, and ROS while restoring the activities of SOD and GSH in the colons of DSS-treated mice. Further analysis demonstrated that these antioxidant and anti-ferroptotic effects occurred in a dose-dependent manner.

The tumor suppressor p53 is a key stress-response regulator activated by diverse cellular insults [74,75]. In the context of ferroptosis, p53 inhibits the expression of solute carrier family 7 member 11 (SLC7A11), a core component of the cystine/glutamate antiporter system Xc^−^. This suppression restricts cystine uptake, leading to glutathione (GSH) depletion. The subsequent decrease in GSH levels impairs the activity of glutathione peroxidase 4 (GPX4), compromising the cellular antioxidant defense system and promoting ferroptosis [76]. Numerous studies have shown that traditional Chinese medicine and its monomer components can alleviate ulcerative colitis and intestinal damage by inhibiting ferroptosis through regulating these compounds. Pinobanksin is one of the main flavonoids derived from propolis. Bi Hailian et al. demonstrated that pinobanksin ameliorates DSS-induced acute colitis, primarily by modulating SLC7A11/glutathione-mediated ferroptosis in intestinal epithelial cells [77]. Compound sophorae decoction (CSD) has been extensively applied in the clinic for the treatment of ulcerative colitis (UC). In a rat model of colitis, Compound Sophorae Decoction (CSD) was verified to alleviate ferroptosis through the regulation of GPX4 and SLC7A11 [78]. Separately, Huangqin Decoction was found to effectively reduce ferroptosis, inflammation, and oxidative stress related to intestinal injury by modulating the P53/SLC7A11/GPX4 pathway [79]. Consequently, targeting the p53/SLC7A11/GPX4 pathway to inhibit ferroptosis represents a promising therapeutic strategy for UC [80].

Our study confirmed that FF inhibits ferroptosis in colitis. To elucidate its molecular targets, we employed network pharmacology, which identified the main active ingredients of FF and predicted ferroptosis-related targets. Decursin, Imperatorin, and Isoimperatorin were ranked as the top compounds by network centrality. Notably, these compounds have been previously reported to possess anti-inflammatory and antioxidant properties [81,82,83,84], in addition to the ferroptosis inhibitory effect mentioned above. Protein–protein interaction (PPI) network analysis identified p53 as the highest-ranked core target. Molecular docking simulations further supported this finding, showing favorable binding energies between these active compounds and the p53 protein. Enrichment analysis of common targets highlighted the significant involvement of the p53 signaling pathway.

Consistent with its proposed role, immunohistochemistry revealed a marked increase in p53 protein expression alongside decreased levels of SLC7A11 and GPX4 in the colons of DSS-treated mice. FF treatment effectively normalized the expression of all three proteins. These results demonstrate that p53 is a critical ferroptosis-related target through which FF alleviates experimental colitis.

In recent years, ferroptosis has gained increasing recognition as a novel form of regulated cell death implicated in the pathogenesis of inflammatory bowel disease (IBD) [85]. Consequently, the potential of Chinese herbal medicines to protect the intestinal mucosa by modulating ferroptosis has emerged as a promising area of investigation [86]. This study provides novel evidence that *Saposhnikovia divaricata* (FF) ameliorates experimental ulcerative colitis by concurrently attenuating inflammation, oxidative stress, and ferroptosis. Our mechanistic investigation suggests that this protective effect is mediated, at least in part, through the modulation of the p53/SLC7A11/GPX4 signaling axis. These findings identify a new therapeutic target and provide a scientific rationale for the application of FF in UC management.

Furthermore, network pharmacology predictions indicated the potential involvement of additional pathways—including HIF-1, PI3K-Akt, TNF, and NF-κB signaling—in the anti-ferroptotic effects of FF. This suggests that FF alleviates UC via a multi-target mechanism, simultaneously regulating inflammatory, immune, ferroptotic, and metabolic pathways.

While this study provides preliminary evidence that FF inhibits oxidative stress and ferroptosis in a mouse model of UC, several limitations must be acknowledged. The etiopathogenesis of human UC is highly complex and multifactorial, and acute DSS-induced colitis in mice cannot fully recapitulate the chronic, relapsing nature of the human disease. Consequently, the therapeutic characteristics and long-term efficacy of FF in chronic UC remain to be established. Furthermore, the clinical safety and sustained effectiveness of FF require validation through rigorous future clinical trials.

Oxidative stress is a widely studied topic. This experiment was limited to examining oxidative stress markers in inflammation and ferroptosis, without exploring its roles in DNA/RNA/protein damage or apoptosis, which should be addressed in future studies to advance the understanding of oxidative stress. Although we have demonstrated that FF’s anti-ferroptotic effect involves the p53 pathway, the mechanistic evidence remains correlative. To establish a definitive causal relationship, future studies should employ genetic approaches, such as p53 knockdown or knockout in both cellular and animal models. This would allow for a more precise verification of p53′s regulatory role in ferroptosis within the context of UC and FF treatment.

## 5. Conclusions

In this study, we demonstrated that FF ameliorates UC by inhibiting inflammation, oxidative stress and ferroptosis. The results illustrated that FF reduced inflammatory cytokines and the accumulation of ROS. Meanwhile, FF relieved symptoms in UC mice by modulating the levels of oxidative stress and ferroptosis markers. Based on network pharmacology predictions, FF can down-regulate the expression of p53 protein and up-regulate the expression of SLC7A11 and GPX4 proteins. These findings suggest that the protective effect of FF against DSS-induced UC is associated with the p53/SLC7A11/GPX4 pathway.

## Figures and Tables

**Table 1:** Disease activity index (DAI).

Score	Body Weight Loss (a)	Stool Traits (b)	Stool Occult Blood (c)
0	No	Normal	-
1	1%~5%		+
2	6%~10%	Loose stools	+ +
3	11%~15%		+ + +
4	>15%	Watery diarrhea	+ ++ +

**Table 2:** Colonic mucosal damage index (CMDI).

Score	Colon Changes
0	No damage.
1	Mild congestion, edema, smooth surface, no erosion or ulcer.
2	Hemorrhagic edema, coarse granular mucosa, erosion or intestinal adhesion.
3	Highly congestion and edema, mucosal surface necrosis and ulcer formation, ulcer maximum longitudinal diameter < 1 cm, intestinal wall thickening or surface necrosis and inflammation.
4	Maximum longitudinal diameter of ulcer > 1 cm or total intestinal wall necrosis based on 3 points.

**Table 3:** Identification compounds of FF via UPLC-Q-Orbitrap HRMS analysis.

No.	Retention Time (min)	Experimental *m*/*z*	Theoretical *m*/*z*	Mass Error (ppm)	Formula	Compound
1	7.46	353.0875	353.0878	−0.92	C_16_H_18_O_9_	Chlorogenic acid
2	9.75	369.1167	369.1180	−3.66	C_17_H_20_O_9_	4-O-Feruloylquinic acid
3	10.44	469.1685	469.1704	−4.14	C_22_H_28_O_11_	Prim-O-glucosylcimifugin
4	10.84	191.0346	191.0350	−1.77	C_10_H_8_O_4_	Scopoletin
5	11.98	307.1161	307.1176	−4.85	C_16_H_18_O_6_	Cimifugin
6	12.03	409.1478	409.1493	−3.70	C_20_H_24_O_9_	Nodakenin
7	12.35	515.1189	515.1195	−1.20	C_25_H_24_O_12_	Isochlorogenic acid A
8	12.43	565.1924	565.1927	−0.56	C_26_H_32_O_11_	(+)-Pinoresinol 4-O-glucoside
9	12.80	453.1735	453.1755	−4.53	C_22_H_28_O_10_	5-O-Methylvisammioside
10	14.37	445.0774	445.0776	−0.61	C_21_H_18_O_11_	Baicalin
11	14.87	247.0956	247.0965	−3.75	C_14_H_14_O_4_	(±)-Prantschimgin
12	14.91	336.1216	336.1230	−4.31	C_20_H_18_NO_4_+	Berberine
13	15.37	291.1213	291.1227	−4.72	C_16_H_18_O_5_	5-O-Methylvisamminol
14	15.94	483.1499	483.1508	−2.10	C_21_H_26_O_10_	Sec-O-glucosylhamaudol
15	16.44	461.1064	461.1078	−3.14	C_22_H_20_O_11_	Wogonoside
16	16.52	275.0924	275.0925	−0.48	C_15_H_16_O_5_	Hamaudol
17	16.64	187.0384	187.0390	−3.30	C_11_H_6_O_3_	Psoralen
18	17.30	217.0487	217.0495	−3.90	C_12_H_8_O_4_	Methoxsalen
19	18.16	217.0486	217.0495	−4.22	C_12_H_8_O_4_	Bergapten
20	20.77	271.0952	271.0965	−4.73	C_16_H_14_O_4_	Imperatorin
21	21.49	329.1368	329.1383	−4.64	C_19_H_20_O_5_	Decursin
22	21.51	271.0953	271.0965	−4.44	C_16_H_14_O_4_	Isoimperatorin
23	23.58	444.2003	444.2017	−3.23	C_24_H_26_O_7_	Anomalin

**Table 4:** Top 10 key compounds of FF.

No.	Compound	Degree
1	Wogonin	17
2	Decursin	10
3	Isoimperatorin	9
4	Imperatorin	8
5	methyl icosa-11,14-dienoate	8
6	5-O-Methylvisamminol	7
7	Phellopterin	7
8	Phelloptorin	7
9	Anomalin	7
10	Mandenol	6

**Table 5:** Molecular docking results of the top four active compounds and core targets.

Compound	Binding Energy (kcal/mol)
Decursin	−7.463
Imperatorin	−6.205
Isoimperatorin	−5.816

## Data Availability

Data are contained within the article.
